# Poloxamer 407/188 Binary Thermosensitive Gel as a Moxidectin Delivery System: In Vitro Release and In Vivo Evaluation

**DOI:** 10.3390/molecules27103063

**Published:** 2022-05-10

**Authors:** Xiangchun Ruan, Jidong Hu, Lianshou Lu, Youwei Wang, Chunlian Tang, Faquan Liu, Xiuge Gao, Li Zhang, Hao Wu, Xianhui Huang, Qing Wei

**Affiliations:** 1 Laboratory of Veterinary Pharmacology and Toxicology, College of Animal Science and Technology, Anhui Agricultural University, Hefei 230036, China; rxc@ahau.edu.cn (X.R.); hzjqd123@163.com (J.H.); wangyouweiwyw1996@163.com (Y.W.); 2 China Institute of Veterinary Drug Control, Beijing 100081, China; luzhengxin20200202@126.com; 3 Anhui Institute of Veterinary Drug and Feed Control, Hefei 230091, China; tangchunlian2022@163.com (C.T.); sjj66138@163.com (F.L.); zhangli9400@126.com (L.Z.); whahdj@163.com (H.W.); 4 Laboratory of Veterinary Pharmacology and Toxicology, College of Veterinary Medicine, Nanjing Agricultural University, Nanjing 210095, China; vetgao@njau.edu.cn; 5 Laboratory of Veterinary Pharmacology and Toxicology, College of Veterinary Medicine, South China Agricultural University, Guangzhou 510642, China; xhhuang@scau.edu.cn; 6 College of Eco-Environmental Engineering, Qinghai University, Xining 810016, China

**Keywords:** moxidectin, thermosensitive gel, in vitro release, in vivo evaluation, Qinghai Tibetan sheep

## Abstract

Moxidectin (MXD) is an antiparasitic drug used extensively in veterinary clinics. In this study, to develop a new formulation of MXD, a thermosensitive gel of MXD (MXD-TG) was prepared based on poloxamer 407/188. Furthermore, the gelation temperature, the stability, in vitro release kinetics and in vivo pharmacokinetics of MXD-TG were evaluated. The results showed that the gelation temperature was approximately 27 °C. MXD-TG was physically stable and can be released continuously for more than 96 h in vitro. The Korsmeyer–Peppas model provided the best fit to the release kinetics, and the release mechanism followed a diffusive erosion style. MXD-TG was released persistently for over 70 days in sheep. Part of pharmacokinetic parameters had a difference in female and male sheep (*p* < 0.05). It was concluded that MXD-TG had a good stability, and its release followed the characteristics of a diffusive erosion style in vitro and a sustained release pattern in vivo.

## 1. Introduction

Parasitism is one of the most common infections in livestock. Especially, gastrointestinal nematode (GIN) infections impact directly and indirectly on animals as well as on the associated economic production. In sheep, GIN can produce anemia, diarrhea and severe protein loss. In addition, parasitism can have indirect consequences on the metabolism by an increased susceptibility to other pathogens [1,2,3]. Benzimidazoles, salicylanilides, imidazothiazoles and macrocyclic lactones are widely used to control GIN [4]. However, the frequent use of the same drug causes resistance to the main anthelmintic used [5,6]. This favors GIN infestations, affecting the performance of the sheep and decreasing the productive efficiency and economic development of the flock [7].

Moxidectin (MXD) is an effective broad-spectrum insectifuge [8] and is widely used in a variety of mammalians for controlling nematodes and mites [9,10,11]. Compared to ivermectin, MXD has a better curative effect, higher distribution, longer elimination half-life and better safety [12]. MXD had the shortest recovery time and completely eliminated parasitic worm eggs in feces in buffalo calves infected with *toxoplasma gondii*. It has been indicated that MXD has a stronger insecticidal activity than IVM and piperazine citrate [13]. Di Cesare [14] evaluated the efficacy and safety of 10% imidacloprid +1% MXD on cats with a natural infection of *Capillaria aerophile*. The deworming rate was 100% in the treatment group, and no adverse reactions occurred during the experiments. At present, the common formulations of moxidectin in clinics include injections, tablets and transdermal agents, which are applied to animals such as cattle, sheep, horses and pigs, and are ideal for the internal and external antiparasitic. A total of 2% for sheep and 10% for cattle of moxidectin injections (Cydectin^®^) have been authorized in the European Union.

A thermosensitive gel (TG) system can be fabricated from natural or synthetic materials, which are typically biocompatible and degrade into safe byproducts. The gel systems have been developed to address short local retention. The gel system is in the sol form and undergoes gelation in situ after the administration to the body. Gel formation depends on several factors, such as pH change, temperature modulation or the presence of ions. The advantages of the system include prolonged drug delivery, decreased administration times, reduction in side effects and improved animal comfort and compliance. The system combines the benefits of both solutions and gels, which enhances its bioavailability [15,16].

An ideal TG for drug delivery requires a low viscosity solution at room temperature that gels at body temperature. TGs have been largely developed and used for accurate dosing and convenient administration. Poloxamers are used as surfactants as well as gelling agents for the preparation of TGs. Poloxamers, co-polymers composed of polyethylene oxide and polypropylene oxide units, have been investigated as drug delivery systems, showing promising results concerned with the improvement of the biopharmaceutic, pharmacodynamic and pharmacokinetic properties of the incorporated drugs. One of the main advantages of the poloxamers is the capability of forming gels close to body temperature [17]. With good temperature sensitivity and biocompatibility, poloxamer 407 (P407) is an ideal excipient to prepare temperature-sensitive in situ gel, whereas poloxamer 188 (P188) has been added appropriately to modulate the gelation temperature [18].

TGs as drug-loaded carriers provide the sustained drug release of therapeutic agents. When gels act as direct drug carriers, the simplest form involves the suspension of a drug within the carriers, allowing the drug to diffuse out of the gel and into the surrounding space [19,20]. Therefore, this study is conducted to develop an MXD injectable thermosensitive gel (MXD-TG) based on P407/P188 [21]. The gelation temperature, the stability, in vitro release kinetics and in vivo pharmacokinetics of MXD-TG are evaluated.

## 2. Results

### 2.1. Gelation Temperature Determination

The gelation temperature of MXD-TG was approximately 27 °C. Meanwhile, the gelation characterization was observed. MXD-TG was gelated at 2 min, which was transferred to a small beaker and incubated at 37 °C.

### 2.2. Stability Analysis

The calibration curve of MXD was prepared by determining the best fit of peaks area ratios vs. concentration (1–50 µg/mL), and fitted to Y = 14.178X − 0.2567. There was a good linear relationship (R^2^ = 0.9999). The results indicate that the color of MXD-TG did not change, and stability under the intended packaging after 5 and 10 days placing at the condition of high temperature or strong light. The content of MXD was 103.17 ± 1.41% and 99.22 ± 1.11% at 5 and 10 days at 60 °C, respectively. The content of MXD was 101.68 ± 3.37% and 98.27 ± 3.12% at 5 and 10 days at 4500 Lx light, respectively. Similarly, three batches of MXD-TG were relatively stable at 4 °C and 25 °C for three months. The results are shown in Table 1.

### 2.3. In Vitro Release

#### 2.3.1. In Vitro Release Determination

**Figure 1:**
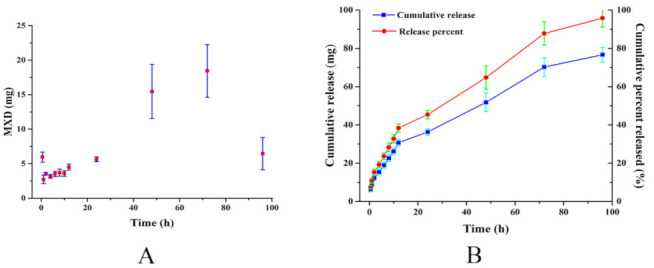
In vitro release of MXD-TG. (**A**) The release amount of MXD at different times; (**B**) the cumulative release and cumulative percent released of MXD.

There was 5.961 ± 0.725 mg of MXD released from the MXD-TG at half hour. Subsequently, the release of MXD was steady within the range of 3–4 mg during the next 2 h to 10 h. The release amount of MXD was 36.323 ± 1.752 mg at 1 day. Afterwards, the release of MXD was 15.465 ± 3.921 mg and 18.439 ± 3.821 mg from gel at 2 days and 3 days, respectively. Finally, the release amount of MXD decreased to 6.446 ± 2.335 mg at 4 days, and the MXD-TG was almost completely dissolved. A burst effect was observed, with 45.40 ± 2.19% of the MXD released at 1 day, followed by a slower release. The total released amount of MXD was 76.67 ± 3.87 mg, corresponding to 95.84 ± 4.84% of the initial content. The in vitro release of MXD-TG is shown in Figure 1.

#### 2.3.2. Release Kinetic Models and Release Mechanism

Different release kinetics models were simulated. The Korsmeyer–Peppas model provided the best fit to the dissolution release kinetics, exhibiting an R^2^ value (R^2^ = 0.9917) close to 1 and the lowest Akaike Information Criterion (AIC, AIC = 54.83). The release mechanism is further explained by the parameter n of the Korsmeyer–Peppas model. The value of n was 0.493, indicating the release of MXD occurs through a diffusive erosion style [22]. The calculated R^2^ and AIC parameters corresponding to each model are shown in Table 2.

### 2.4. In Vivo Evaluation

#### 2.4.1. HPLC-MS/MS Method Validated

HPLC-MS/MS assay method for MXD was validated. The analytical curve prepared by the addition of MXD to the blank plasma was linear in the range of 0.5–200.0 ng/mL (Y = 14.768X − 0.4774, R^2^ = 0.9969). The limits of detection and quantification of MXD were 0.2 ng/mL and 0.5 ng/mL, respectively. The recovery of MXD was >94% at 0.5, 1, 10, 100 ng/mL with six parallel samples. The intra-day and inter-day precisions were <3.95% and <3.61% at 0.5, 1, 10, 100 ng/mL with six parallel samples, respectively.

#### 2.4.2. Blood Concentration of MXD in Qinghai Tibetan Sheep

**Figure 2:**
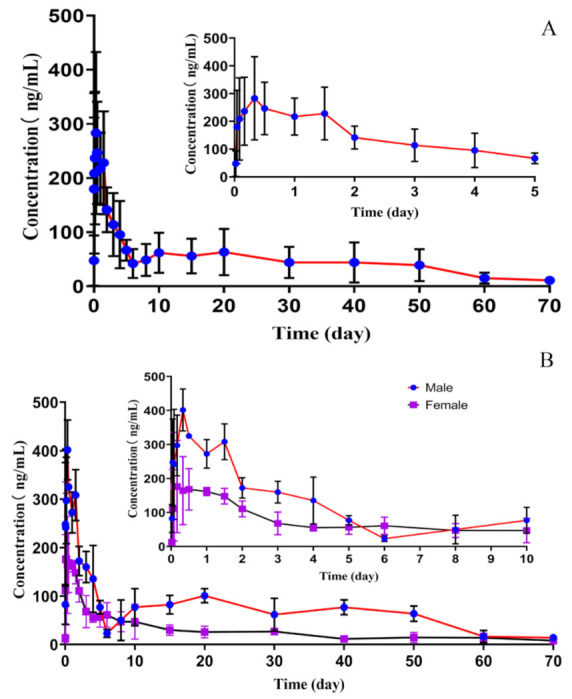
The concentration of MXD in the plasma of Qinghai Tibetan sheep. (**A**) The concentration of MXD in the plasma of Qinghai Tibetan sheep within 70 days. (**B**) The concentration of MXD in the plasma of male and female Qinghai Tibetan sheep within 70 days.

The Qinghai Tibetan sheep had no side effects during the experiment. The MXD-TG exhibited sustained release for over 70 days in vivo after subcutaneous administration. MXD was absorbed quickly and the concentration of MXD was 47.67 ± 42.43 ng/mL at the half hour mark. Afterward, the concentration of MXD in the plasma increased gradually and was still over 100 ng/mL at 3 days, and remained relatively stable from 5 to 50 days. Then, the concentration of MXD decreased to 15.15 ± 9.19 and 11.12 ± 5.89 ng/mL at 60 days and 70 days, respectively (Figure 2A). Meanwhile, the concentration of MXD in male sheep was high in comparison to that in female sheep, except at six days (Figure 2B).

#### 2.4.3. Pharmacokinetics

The C_max_, T_max_ and T_1/2β_ were 323.49 ± 128.57 ng/mL, 0.39 ± 0.34 day and 16.22 ± 6.66 days, respectively. The AUC_0_–_t_ and AUC_0_–_∞_ were 3285.56 ± 1582.40 day·ng/mL and 3552.41 ± 1657.46 day·ng/mL. The V_d_ and CL were 8732.96 ± 6207.47 mL/kg and 346.70 ± 171.93 mL/day/kg. The MRT_0_–_t_ and MRT_0_–_∞_ were 23.22 ± 3.95 days and 29.13 ± 3.67 days. The difference of pharmacokinetic parameters was present in male and female Qinghai Tibetan sheep. The AUC_0_–_t_ and AUC_0_–_∞_ were 4690.86 ± 472.67 and 5008.83 ± 525.14 day·ng/mL in male sheep, 1880.26 ± 334.62 and 2095.99 ± 478.20 day·ng/mL in female sheep, respectively. The AUC_0_–_t_ and AUC_0_–_∞_ in male sheep were higher than those in female sheep, and had a significant difference (*p* < 0.01). However, the CL in male sheep was lower than that in female sheep, and had a significant difference (*p* < 0.01). Similarly, the V_d_ in male sheep had a significant difference in female sheep (*p* < 0.05). The differences in C_max_, T_max_, MRT and T_1/2β_ between male and female sheep had no statistical significance (*p* > 0.05) (Table 3).

## 3. Discussion

It has been reported that the optimal P407 concentration in in situ gel systems is approximately 20% [23]. The addition of a drug and other excipients can change gelation temperature and gel viscosity. To increase cementitious capability, we added gelled biomaterials to the formulation, including neutral polymer methyl cellulose (MC) and P188. The gelation temperature of the 22% P407 solution changed evidently after adding 1% P188. By contrast, an increase in MC or MXD did not significantly affect the gelation temperature. Drug release can be changed by modulating various composition parameters, such as polymer materials, polymer concentration and sustained-release agents [24,25]. Correspondingly, the varying gel degradation rate can be used to modulate the drug release rate [26]. The MC was added as a sustained-release agent to gel. The release time of MXD-TG containing MC was 24 h longer than that of MXD-TG without MC in vitro. Meanwhile, MC can also play a role as a suspending agent and reduce the settling velocity of MXD in gel solutions.

A type of composite TG involves the dispersion of drug particles into gel matrices. This approach can be utilized to provide the sustained release of sparingly soluble drugs. A thermoresponsive poloxamer (P407)-polyvinyl alcohol gel was developed to deliver mupirocin nanoparticles for wound healing [27]. Silver sulfadiazine/nanosuspensions (AgSD/NS) were prepared and loaded into poloxamer 407-based TGs as carriers of AgSD/NS to obtain AgSD/NS-loaded TGs [28]. In this study, MXD particulates were prepared by the homogenization method. A poloxamer (P407/188)-based TG was prepared by the cold method to obtain injectable MXD-TG. The stability results show that the MXD-TG in the intended packaging was less affected by the high temperature and strong light, indicating that the MXD-TG had a good stability. Meanwhile, the MXD-TG was stable at 4 °C and 25 °C for three months too. 

Moxidectin is insoluble in water and soluble in ethanol, dichloromethane, and acetonitrile, etc. In order to fully dissolve MXD into the release medium after the release from the TG, a certain volume fraction of ethanol was added to the release medium. Polyoxyethylene (20) Oleyl Ether (Brij^®^ 98, POE) is a surfactant similar to sodium dodecyl sulfate, POE, which increases the solubility of MXD in aqueous solutions. The release medium composed of ethanol, POE and water can satisfy with the sink condition of MXD release in vitro. A membraneless dissolution method was used for the MXD-TG release in vitro [29]. Although it can not completely simulate the release in vivo, it is used in in vitro release studies because of its simplicity and convenience. When gels act as drug delivery carriers, the particles of MXD were dispersed in the TG, allowing the MXD to diffuse out of the gel and into the surrounding space. In present study, about 45% of the MXD content was released at the first day from the MXD-TG in vitro and exhibited sustained release for over 96 h. It was showed that the release of MXD from the gel was slow with burst effects at an early stage. Due to the difference of release environment between in vitro and in vivo, the release time of MXD-TG in vitro was much shorter than that in vivo.

In addition, the establishment of an appropriate drug release model, through the fitting of the experimental data, was an effective means to simulate and predict the release behavior of MXD-TG. It was reported that the hydrogel-loaded mupirocin nanoparticles fitted the first-order kinetics, which showed that the drug release was controlled mainly by diffusion [27]. Differently, the gemcitabine release of high-molecular-weight hyaluronic-acid-added gel was diffusion-erosion controlled [30]. The injectable TG combined with dimethoxycurcumin showed a Korsmeyer–Peppas-model-fitted sustained-release behavior [31]. In this study, methyl cellulose was selected as the sustained release agent to prolong the release time of MXD-TG in vitro. The Korsmeyer–Peppas model provided the best fit to the dissolution kinetics data, as it produced an R^2^ value close to 1 and the lowest AIC among the tested models. Moreover, the n parameter of the Korsmeyer–Peppas model was 0.493. If n < 0.45, this situation is called “less Fickian” behavior. It is a diffusion type with an erosion rate larger than the diffusion rate, and the equilibrium swelling value is reached in a short time. If n = 0.45, this situation is called Fickian diffusion. Polymer chains have high mobility, and water easily permeates through the network structure. If n > 0.45, this situation is a non-Fickian type or abnormal solvent diffusion type. There is insufficient motion to ensure water permeates into the interior of the polymer in the polymer chains. In other words, during the swelling of the gel, diffusion and erosion occur at the same time [22]. So, the release of MXD occurs through a diffusive erosion mechanism.

In in vivo studies, the C_max_/dose and AUC_0_–_t_/dose of the MXD-TG preparation were 323.49 ± 128.57 ng mL^−1^ mg^−1^ kg and 3285.56 ± 1582.40 days ng mL^−1^ mg^−1^ kg, respectively. The C_max_/dose and AUC_0_–_t_/dose of the commercial MXD formulation was 42 ± 28.5 ng mL^−1^ mg^−1^ kg and 1094 ± 188.5 days ng mL^−1^ mg^−1^ kg [32], respectively. It was shown that MXD could be absorbed quickly and distributed widely after subcutaneous administration. Moreover, the MXD-TG exhibited a sustained release for over 70 days in Qinghai Tibetan sheep. The concentration of MXD remained relatively stable in the range of 5–50 days and was still above 10 ng/mL in the plasma at 70 days. Comparing with the oral formulation, the concentration of MXD was too low to be detected in the plasma after 42 days of administration at 0.2 mg/kg [33]. Meanwhile, the MXD concentration remained above 1 ng/mL when treated with a subcutaneous administration at 0.2 mg/kg during the 40 days of the experiment period [32]. The maintenance time of MXD was shorter than that of the MXD-TG preparation in the plasma of sheep. Despite differences in administration and dosage, the MXD-TG exhibited sustained-release properties. It is inferred that MXD-TG can maintained its antiparasitic efficacy for more than 70 days after subcutaneous administration.

The pharmacokinetic parameters of MXD were described in both groups of ewes after subcutaneous administration at 0.2 mg/kg. The values of C_max_, T_max_ and AUC were similar for both groups of ewes, and no significant differences were observed. However, the values for the terminal half-life and MRT observed in the pregnant ewes were significantly lower than those observed in the control nonpregnant sheep [32]. Similarly, a difference was observed in MXD pharmacokinetics in male and female beagle dogs [34]. In this study, part of the pharmacokinetic parameters of the MXD-TG had a statistical difference in male and female sheep. The AUC in male sheep was higher than in female sheep and had a significant difference (*p* < 0.01). Conversely, the V_d_ and CL in female sheep were higher than in male sheep. Within male and female sheep, the V_d_ had a significant difference (*p* < 0.05) and CL had an extremely significant difference (*p* < 0.01). The difference in MXD distribution may be related to the difference in fat distribution between males and females [35]. It can be inferred that the pharmacokinetic parameters of MXD are affected by the sexual, physiological and infection status of the animal [12].

## 4. Materials and Methods

### 4.1. Reagents and Chemicals

MXD (moxidectin, ≥95.5%) was donated by Xinyu Pharmaceutical Co., Ltd. (Suzhou, China). Poloxamer 407 and Poloxamer 188 were purchased from Guangzhou Ruixin Chemical Industry Co., Ltd. (Guangzhou, China). Polyoxyethylene (20) Oleyl Ether (Brij^®^ 98, CAS:9004-98-2) was produced by Sigma-Aldrich (Shanghai, China). All the solvents and reagents used in the extraction procedure were of HPLC grade or analytical grade. Ultra-pure water was obtained from a Milli-Q pure water system (Millipore, Burlington, MA, USA).

### 4.2. Apparatus

Agilent HPLC system was composed of an Agilent Infinity 1260 quaternary pump, an autosampler injector, a column oven and an ultraviolet detector (Agilent, Valtbrone, Germany). Chromatographic separation was achieved with Agilent Exctend-C_18_ column (150 mm × 4.6 mm, 5 µm). HPLC-MS/MS were comprised with an Agilent 1290 Infnity II liquid chromatograph coupled to an Agilent 6470 triple quadrupole mass spectrometer (Agilent, Santa Clara, CA, USA). Chromatographic separation was achieved with an Agilent ZORBAX Eclipse Plus C_18_ column (50 mm × 2.1 mm, 8 µm, Agilent, Santa Clara, CA, USA).

### 4.3. Preparation of MXD-TG 

The MXD-TG was prepared using the cold method [18]. Briefly, MXD was added into a proper amount of pure water and homogenized for 10 min at 30,000 rpm (T10, IKA, Staufen, Germany). Then, the poloxamer 407 and poloxamer 188 were dispersed in the previously prepared MXD suspensions. The dispersion was kept in a refrigerator at 4 °C overnight until the entire poloxamer was dissolved and finally formed clear solution. Then, methyl cellulose was added as a sustained-release agent to the solution and it was mixed thoroughly. Finally, pure water was added to the constant volume. The formulation was kept in a refrigerator prior to evaluation. The prepared formula’s batch size was one liter. The composition of the gel, including the exact amounts of active ingredients and excipients, are shown in Table 4.

### 4.4. Gelation Temperature Measurement

The tube inversion method was used to determine the gelation temperature of several thermosensitive gels. The gelation temperature of the MXD-TG was determined using the method reported previously [18]. Briefly, four grams of the MXD-TG solution was transferred to tubes and incubated in a water bath and equilibrated at 15 °C for 10 min. The sol–gel transitions of the solutions were evaluated by the tube inversion method at a temperature range of 15–45 °C with a heating rate of 0.5 °C per min. The gelation temperature was defined as the temperature at which the sample did not flow following the inversion of the test tubes. The tests were repeated three times. Meanwhile, for in vitro gelation characterization, 10.0 g of MXD-TG was transferred to a small beaker and incubated at 37 °C. The system was monitored every 1 min for gelation.

### 4.5. Stability Tests

The MXD-TG was packed in the brown glass sample bottle, and tightly sealed with a lid containing a gasket inside. Then, the sample bottles were placed in a drug stability tester (WD-A, Tianjin pharmacopoeia standard instrument factory, Tianjin, China). The affection was evaluated in 60 ± 2 °C or 4500 ± 500 Lx light at 0, 5 and 10 days. The sample was six replicates as parallel samples. Meanwhile, the MXD-TGs were stored at 4 °C and 25 °C for three months. The stability was investigated at 0, 1, 2 and 3 months. The sample was three replicates as parallel samples. The change in MXD content was analyzed referring the method of European pharmacopoeia 8.0 with slight modifications. Briefly, the calibration curve of MXD at 1–50 µg/mL was prepared. The samples were diluted with methanol to a concentration of about 20 µg/mL for MXD and filtered through 0.45 µm nylon syringe filter (Pilot, Tianjin, China) into HPLC vials [36]. The adequate separation of the analyte was achieved by HPLC (1260 series, Agilent, Santa Clara, CA, USA) with Agilent Exctend-C_18_ column (150 mm × 4.6 mm, 5 µm) at 35 °C. The mobile phase was an ammonium acetate buffer solution (pH = 4.2)–acetonitrile (25:75, *v*/*v*) at a flow rate of 2.0 mL/min and the wavelength of detection was at 242 nm.

### 4.6. In Vitro Studies 

#### 4.6.1. In Vitro Release 

A membraneless dissolution method was used for the in vitro release in MXD-TGs [29]. A total of 4.0 g of MXD-TG was added in a 15 mL tube with a stopper, which was placed in a water bath at 37 °C for ten minutes until completely gelated. The release medium was warmed beforehand in a water bath at 37 °C. The release medium contained POE (6%), ethanol (40%) and Ultrapure water (54%), which satisfied the sink conditions of MXD in in vitro release. Additionally, 8 mL of the release medium was added gently into the tube, which was placed in the water bath constant temperature oscillator (SHA-C, Ronghua, Wuxi, China) at 37 °C with shaking at 100 r/min. The release medium was taken out completely at 0.5, 1, 2, 4, 6, 8, 10, 12, 24, 48, 72 and 96 h, and collected into a centrifugal tube after measuring the volume. The tube was supplied with 8 mL of release medium and the above operation was repeated until the gel was almost completely released. The samples were diluted with methanol to a concentration of 1–50 µg/mL for MXD and filtered through a 0.45 µm nylon syringe filter (Pilot, Tianjin, China) into HPLC vials. The concentration of MXD was detected using the above HPLC method. The MXD content in the release medium was the concentration of MXD determined by HPLC times the volume of the release medium. The cumulative release and cumulative percent released of MXD was calculated.

#### 4.6.2. In Vitro Release Properties

The properties of the MXD-TG in vitro release were kinetically simulated using mathematical models. Drug release kinetics, using a model dependent on DD Solver software, were used for the analysis of the following: zero order, first order, Higuchi, Korsmeyer–Peppas, etc. [37].

### 4.7. In Vivo Evaluation

#### 4.7.1. Animal Experiment

Six Qinghai Tibetan sheep (three male and three female) were selected with a body weight of 27.98 ± 1.78 kg. A single dose of MXD-TG was administrated by a subcutaneous injection at 1 mg/kg·bw after fasting 12 h. Blood samples were collected at 0, 0.5, 1, 2, 4, 8, 12, 24, 36 h and 2, 3, 4, 5, 6, 8, 10, 15, 20, 30, 40, 50, 60 and 70 d. The samples were centrifuged at 1801× *g* (SC-3614, Zonkia, Hefei, China) for 10 min and the plasma was stored at −18 °C until the analysis was carried out. The experiment was conducted in accordance with the animal welfare regulations of the animal ethics committee of Qinghai University.

#### 4.7.2. Sample Preparation and Analysis

A total of 200 µL of plasma was added with 1 mL of acetonitrile and the sample was mixed by a vortexer for 2 min, and centrifugated at 12,830× *g* (HC-2518R, Zonkia, Hefei, China) for 10 min at 8 °C. The supernatant was transferred into 2 mL centrifugal tube and dried under a stream of nitrogen at 50 °C (EFAA-DC24-RT, Anpel, Shanghai, China). The dried residue was dissolved in acetonitrile (1 mL), and centrifugated at 12,830× *g* for 5 min at 8 °C after vortexing for 2 min. The sample was transferred into a vial after filtering through a 0.22 µm nylon syringe filter (Pilot, Tianjin, China) for analysis using HPLC MS/MS. The MXD was detected using the HPLC-MS/MS method with slight modifications [38]. The chromatographic separation was carried out using an Agilent Eclipse Plus C_18_ column at 40 °C. An injection volume of 5 µL was used, the mobile phase flow rate was 0.4 mL/min and the run time was 5 min. The mobile phase was composed of acetonitrile (mobile phase A) and 0.1% formic acid aqueous solution (mobile phase B). Gradient elution was applied to separate the analytes. The composition of the mobile phase A was 50% at 0–0.25 min, 50–95% at 0.25–3.0 min and 95–50% at 3.0–5.0 min; the mobile phase B was 50% at 0–0.25 min, 50–5% at 0.25–3.0 min and 5–50% at 3.0–5.0 min. The mass spectrometry parameters were as follows: a drying gas flow of 5 L/min, a drying gas temperature of 300 °C, a sheath gas flow of 11 L/min, a sheath gas temperature of 250 °C, a capillary voltage of 2.50 kV, a nebulized pressure of 45 psi and a nozzle voltage of 500 V. The sample analysis was carried out in multiple reaction monitoring (MRM) in the positive electrospray ionization mode. The precursor ion monitored was *m*/*z* 640.5 and two transitions were selected, as the quantifier ion (*m*/*z* 528.5) and qualifier ion (*m*/*z* 478.5). Data were analyzed using Agilent MassHunter Workstation version B.07 software (Agilent, Santa Clara, CA, USA).

#### 4.7.3. In Vivo Release and Pharmacokinetics 

The in vivo release of MXD-TG in sheep was determined by HPLC MS/MS detection. The blood drug concentration curve was drawn according to the concentration of MXD in the plasma measured at different times. The non-compartmental pharmacokinetic parameters of MXD were analyzed using Phoenix WinNonlin 5.2 (Pharsight Corporation, Mountain View, Santa Clara, CA, USA) software.

### 4.8. Statistical Data Analysis

The results were reported as a means ± standard deviation. Statistical analysis was performed using a one-way ANOVA of SPSS 26.0 software (SPSS Inc., Chicago, IL, USA). The level of significance was accepted at *p* < 0.05.

## 5. Conclusions

A simple, cheap, stable and sustained-release MXD-TG formulation was prepared. MXD-TG was stable under the conditions of high temperature, strong light, 4 °C and 25 °C. The in vitro release kinetics provided the best fit to the Korsmeyer–Peppas equation, and the release mechanism followed a diffusive erosion style. MXD-TG can be absorbed quickly and exhibited a sustained-release in Qinghai Tibetan sheep.

## Figures and Tables

**Table 1:** The stability of the MXD-TG at 4 °C and 25 °C for three months (%).

Condition	Batch	Time (Months)		
		1	2	3
	1	101.30 ± 1.47	101.18 ± 0.94	102.64 ± 4.70
4 °C	2	101.34 ± 2.50	100.58 ± 2.98	100.27 ± 3.24
	3	100.81 ± 0.72	101.00 ± 1.51	99.28 ± 2.75
	1	101.04 ± 1.23	101.40 ± 1.24	100.21 ± 3.71
25 °C	2	101.99 ± 0.49	101.52 ± 1.19	100.40 ± 1.16
	3	102.55 ± 0.45	99.98 ± 0.62	99.76 ± 1.12

Note: The content percentage of MXD at 1, 2 and 3 months was compared with that at 0 month.

**Table 2:** Release kinetics model fitting.

Model	Equation	R^2^	AIC
Zero-order	Q = 1.190 × t	0.6898	97.43
First-order	Q = 100 × [1 − Exp(−0.032 × t)]	0.9251	79.89
Higuchi	Q = 9.895 × t^0.5^	0.9888	56.37
Korsmeyer–Peppas	Q = 10.226 × t^0.493^	0.9917	54.83
Hixson–Crowell	Q = 100 × [1 − (1−0.008 × t)^3^]	0.8897	84.44
Weibull	Q = 100 × {1 − Exp[−((t + 4.22)^0.977^)/49.783]}	0.9805	68.07
Probit	Q = 100 × Φ[−1.826 + 1.426 × log(t)]	0.9438	79.05
Gompertz	Q = 100 × Exp{−5.217 × Exp[−1.602 × log(t)]}	0.9100	87.76

**Table 3:** Pharmacokinetic parameters of MXD obtained from male and female Qinghai Tibetan sheep.

Parameters	Male	Female
T_1/2β_ (day)	13.08 ± 8.10	19.35 ± 3.97
T_max_ (day)	0.24 ± 0.14	0.53 ± 0.46
C_max_ (ng/mL)	415.19 ± 58.93	231.80 ± 112.39
AUC_0_–_t_ (day·ng/mL)	4690.86 ± 472.67 **	1880.26 ± 334.62
AUC_0_–_∞_ (day·ng/mL)	5008.83 ± 525.14 **	2095.99 ± 478.20
V_d_ (mL/kg)	3729.00 ± 2077.32 *	13736.91 ± 4110.70
CL (mL/day/kg)	201.18 ± 21.90 **	492.22 ± 99.44
MRT_0-t_ (day)	25.22 ± 0.79	21.22 ± 5.14
MRT_0-∞_ (day)	29.6 ± 3.56	28.66 ± 4.52

Note: T_1/2β_ = Terminal elimination half-life; T_max_ = Time to peak plasma concentration; C_max_ = Peak plasma concentration; AUC = Area under the concentration–time curve; V_d_ = Apparent volume of distribution; CL = Total body clearance; MRT = Mean residence time. * *p* < 0.05, ** *p* < 0.01.

**Table 4:** The composition of the MXD-TG ingredients.

	% *w*/*v*
Moxidectin	2
Poloxamers 407	22
Poloxamers 188	1
Methyl cellulose	2
Pure water	q.v.

Note: q.v. means quantitative volume.

## Data Availability

Data is contained within the article.

## Sample Availability

Samples of the MXD formulation are available from the authors.
